# [18F]-Fluorodeoxyglucose Positron Emission Tomography Can Contribute to Discriminate Patients with Poor Prognosis in Hormone Receptor-Positive Breast Cancer

**DOI:** 10.1371/journal.pone.0105905

**Published:** 2014-08-28

**Authors:** Sung Gwe Ahn, Minkyung Lee, Tae Joo Jeon, Kyunghwa Han, Hak Min Lee, Seung Ah Lee, Young Hoon Ryu, Eun Ju Son, Joon Jeong

**Affiliations:** 1 Department of Surgery, Gangnam Severance Hospital, Yonsei University College of Medicine, Seoul, Korea; 2 Department of Nuclear Medicine, Gangnam Severance Hospital, Yonsei University College of Medicine, Seoul, Korea; 3 Biostatistics collaboration unit, Gangnam Medical Research Center, Gangnam Severance Hospital, Yonsei University College of Medicine, Seoul, Korea; 4 Department of Radiology, Gangnam Severance Hospital, Yonsei University College of Medicine, Seoul, Korea; 5 Department of Surgery, Eulji University College of Medicine, Seoul, Republic of Korea; University of Texas MD Anderson Cancer Center, United States of America

## Abstract

**Background:**

Patients with hormone receptor-positive breast cancer typically show favorable survival. However, identifying individuals at high risk of recurrence among these patients is a crucial issue. We tested the hypothesis that [^18^F]-fluorodeoxyglucose positron emission tomography (FDG-PET) scans can help predict prognosis in patients with hormone receptor-positive breast cancer.

**Methods:**

Between April 2004 and December 2008, 305 patients with hormone receptor-positive breast cancer who underwent FGD-PET were enrolled. Patients with luminal B subtype were identified by positivity for human epidermal growth factor receptor-2 (HER2) or high Ki67 (≥14%) according to criteria recently recommended by the St. Gallen panelists. The cut-off value of SUV_max_ was defined using the time-dependent receiver operator characteristic curve for recurrence-free survival (RFS).

**Results:**

At a median follow up of 6.23 years, continuous SUV_max_ was a significant prognostic factor with a hazard ratio (HR) of 1.21 (*p* = 0.021). The cut-off value of SUV_max_ was defined as 4. Patients with luminal B subtype (*n* = 82) or high SUV_max_ (*n* = 107) showed a reduced RFS (*p* = 0.031 and 0.002, respectively). In multivariate analysis for RFS, SUV_max_ carried independent prognostic significance (*p* = 0.012) whereas classification with immunohistochemical markers did not (*p* = 0.274). The Harell *c*-index was 0.729. High SUV_max_ was significantly associated with larger tumor size, positive nodes, HER2 positivity, high Ki67 (≥14%), high tumor grade, and luminal B subtype.

**Conclusions:**

Among patients with hormone receptor-positive breast cancer, FDG-PET can help discriminate patients at high risk of tumor relapse.

## Introduction

In patients with hormone receptor (HR)-positive breast cancer, which accounts for approximately 75% of breast cancers [1], endocrine therapies targeting the estrogen receptor (ER) or estrogen synthesis have reduced annual recurrences by 41% and deaths by 34%. Nevertheless, treatment failure still occurs in 30% of patients who are treated with tamoxifen [2], therefore identifying patients with a poor prognosis among those with HR-positive breast cancer has become a critical issue in clinical field. Recent advances in molecular studies based on gene expression profiling have classified subtypes of breast cancer and suggest that HR-positive breast cancer is a clinically and biologically heterogeneous entity [3], [4]. These studies identified at least two major groups of HR-positive tumors, known as luminal A and B. Identification of luminal B tumors among HR-positive cancers using immunohistochemical (IHC) markers has become accepted in clinical practice [5], [6], however, debate against current classification based on IHC markers still remains.

[^18^F] fluorodexoyglucose positron emission tomography (FDG-PET) is a well-established imaging tool for the diagnosis and staging of various malignancies [7]. In addition, the degree of FDG uptake can reflect the biologic characteristics of a tumor and is a validated prognostic factor in various malignancies [8], [9]. In breast cancer, it is well known that ER-positive tumors are characterized by rather low FDG uptake. Previous studies suggest that high FDG uptake is correlated with negative estrogen receptor (ER) expression, negative progesterone receptor (PR) expression, positive human epidermal growth factor receptor-2 (HER-2) expression, high histologic grade, and high proliferative marker such as Ki67 [10]–[12].

Therefore, among ER-positive breast cancer, tumors with increased FDG uptake can be considered to show more aggressive behavior than tumors with decreased uptake. Here, we tested the hypothesis that FDG-PET can help predict prognosis in patients with hormone receptor-positive breast cancer.

## Materials and Methods

### Patient selection

Between January 2004 and December 2008, 835 consecutive women underwent surgery for breast cancer. Of these 835 patients, 682 had undergone preoperative FDG-PET. Patients were excluded on the basis of the following criteria: known bilateral breast cancer (n = 28); preoperative chemotherapy, because chemotherapy can affect tumor characteristics related with FDG uptake (n = 65); ductal carcinoma in situ (n = 83); and distant metastases at initial assessment (n = 41). Among these patients, 309 women with ER-positive and/or PR-positive tumors were identified.

These HR-positive patients were classified as two intrinsic subtypes according to criteria recently recommended by the St. Gallen panelists [5], [6]. The Ki67 cut-off value of 14% also adhered to these criteria. Two subtypes were defined as follows: luminal A (ER-positive and/or PR-positive, HER2-negative and Ki-67<14%); or luminal B (ER-positive and/or PR-positive, HER2-negative, and Ki-67≥14%, or ER-positive and/or PR-positive and HER2-positive, irrespective of Ki67 index). Patients missing data for any IHC marker were excluded (n = 2). Patients with an IHC score of 2+ for HER2 but without fluorescence in situ hybridization (FISH) results for HER2 amplification were also excluded (n = 2). Data for the remaining 305 patients were entered into the analysis (Figure S1).

For IHC evaluation of four markers, formalin-fixed paraffin-embedded tissue sections obtained from surgical specimens were stained with appropriate antibodies for ER (Novocastra, Newcastle upon Tyne, UK), PR (Novocastra), HER2 (Ventana Medical Systems, Tucson, AZ, USA), and Ki-67 (MIB-1; Dako, Glostrup, Denmark). ER and PR were determined by nuclear staining, which was graded from 0 to 8 using the Allred score [13]. The results were categorized as positive when the total score, expressed as the sum of the proportion score and intensity score, was 3 or greater. For HER2 evaluation, membranous staining was graded as 0, 1+, 2+, or 3+ [14]. HER2 status was deemed to be positive for a score of 3+ and negative for a score of 0 or 1+. Tumors with a score of 2+ were sent for FISH analysis using the PathVysion HER2 DNA Probe Kit (Abbott-Vysis, Des Plaines, IL, USA).

Staging was performed according to the American Joint Committee on Cancer (AJCC), 7th edition. The modified Scarf-Bloom-Richardson grading system was used for tumor grading. Adjuvant systemic therapy and/or radiotherapy were considered according to the standard guidelines based on patient age, primary tumor characteristics, and axillary lymph node status. Endocrine therapy was delivered to all patients. The institutional review board (IRB) of Gangnam Severance Hospital, Yonsei University, Seoul, Korea, approved the study in accordance with good clinical practice guidelines and the Declaration of Helsinki. The IRB waived the need for written informed consent from the participants because of the retrospective design.

### FDG-PET method

Prior to FDG-PET, each patient fasted for a minimum of 8 hours and blood glucose level was controlled to lower than 130 mg/dl. Patients received an intravenous injection of 0.14 mCi MBq ^18^F-FDG in the arm contralateral to the primary tumor. At 60 min after injection of ^18^F-FDG, whole-body emission scans were obtained using a Philips Allegro PET camera (Philips Medical Systems, Cleveland, Ohio, USA). All patients were studied in the supine position with their arms raised. Attenuation-corrected transaxial images were reconstructed with an iterative transmission algorithm called a row-action maximum likelihood 3D protocol using a 3D image filter into a 128×128 matrix. For semi-quantitative evaluation, SUV_max_ was calculated by measuring the absorption of 18F-FDG by tumors in the region of interest (ROI) as follows:

**SUV_max_** = [maximal radioactivity concentration in ROI (µCi/g)/injected dose (µCi)/patient’s weight (kg)].

### Statistical analysis

Age was presented as median value with range and compared by Mann–Whitney U test. Discrete variables were compared by chi-square test. The cut-off point of SUV_max_ was defined using the time-dependent ROC curve for recurrence-free survival (RFS).

The primary end-point was RFS. RFS was measured from the date of the first curative surgery to the date of the first loco-regional recurrence or distant metastasis. The Kaplan-Meier method was used to estimate the RFS, and the estimated survival curves were compared using the log-rank test. Significant prognostic factors associated with recurrence-free survival were selected using Harrell *c*-statistic [15] and a Cox proportional hazard regression model was applied for multivariate survival analysis. Student’s *t*-tests were conducted to compare SUV_max_ according to subtype or prognostic factors. All analyses were performed using SPSS version 18 (SPSS; Chicago, IL) and R (http://www.r-projet.org). Statistical significance was defined by a *P*-value<0.05 or a 95% confidence interval (CI) that did not include 1.

## Results

### Patients’ characteristics

Table 1 shows patient characteristics according to the intrinsic subtypes (luminal A and luminal B). The two groups did not differ significantly in T stage, N stage, and AJCC stage, representing tumor burden, or in ER and PR status. In contrast, median age of the luminal A subgroup was significantly higher than that of the luminal B subgroup whereas histologic grade was significantly higher in luminal B than in luminal A. Furthermore, median SUV_max_ was significantly higher in luminal B than in luminal A (4.7 vs. 2.6, respectively). There were no significant differences in adjuvant treatments except for adjuvant endocrine treatment. The higher rate of tamoxifen use in the luminal B subgroup suggested that more premenopausal women were classified as luminal B and was concordant with the lower median age of patients with the luminal B subtype.

**Table 1:** Baseline characteristics of patients according to intrinsic subtypes.

Characteristics	All patients (*n* = 305)	Luminal A (*n* = 223)	Luminal B (*n* = 82)	*P*-value^a^
**Age years, median (range)**	48 (25–80)	48 (28–80)	45 (25–80)	0.019^b^
**T stage**				0.127
T1	174 (57)	135 (60)	39 (48)	
T2	128 (41)	86 (39)	42 (51)	
T3	3 (2)	2 (1)	1 (1)	
**N stage**				0.367
N0	201 (66)	146 (65)	55 (67)	
N1	81 (27)	63 (28)	18 (22)	
N2	17 (5)	11 (5)	6 (7)	
N3	6 (2)	3 (1)	3 (4)	
**AJCC stage**				0.284
I	126 (41)	98 (44)	28 (34)	
II	155 (51)	109 (49)	46 (56)	
III	24 (8)	16 (7)	8 (10)	
**Histologic grade** c				<0.001
1	129 (42)	112 (50)	17 (27)	
2	117 (38)	83 (37)	34 (41)	
3	26 (9)	0 (0)	26 (32)	
**Estrogen receptor**				0.507
Positive	277 (91)	204 (91)	73 (89)	
Negative	28 (9)	19 (9)	9 (11)	
**Progesterone receptor**				0.331
Positive	266 (87)	197 (88)	69 (84)	
Negative	39 (13)	26 (12)	13 (16)	
**HER2**				<0.001
Positive^d^	47 (15)	0 (0)	47 (57)	
Negative	258 (85)	223 (100)	35 (43)	
**Ki67**				<0.001
High	33 (11)	0 (0)	33 (40)	
Low	272 (89)	223 (100)	49 (60)	
**SUV_max_**				<0.001^b^
Median (range)	3.1 (0.8–12.8)	2.6 (0.8–12.8)	4.7 (1.0–11.2)	
**Adjuvant endocrine therapy**				0.030
Tamoxifen	129 (42)	85 (38)	44 (54)	
Toremifene	62 (20)	44 (20)	18 (22)	
Letrozole	61 (20)	49 (22)	12 (15)	
Anastrozole	53 (18)	45 (20)	8 (9)	
**Adjuvant chemotherapy**				0.146
Yes	187 (61)	131 (59)	56 (68)	
No	118 (39)	92 (41)	26 (32)	
**Adjuvant radiotherapy**				0.999
Yes	119 (39)	87 (39)	32 (39)	
No	186 (61)	136 (61)	50 (61)	

AJCC, American Joint Committee on Cancer; SUV_max_, maximum standardized uptake value; HER2, human epidermal growth factor receptor-2.

a Chi-square test, except for ^b^Mann–Whitney U test.

c Data with missing values.

d HER-2 positivity was defined by 3+ score on immunohistochemistry or amplification on fluorescence *in situ* hybridization.

### Survival analysis with SUV_max_

**Figure 1:**
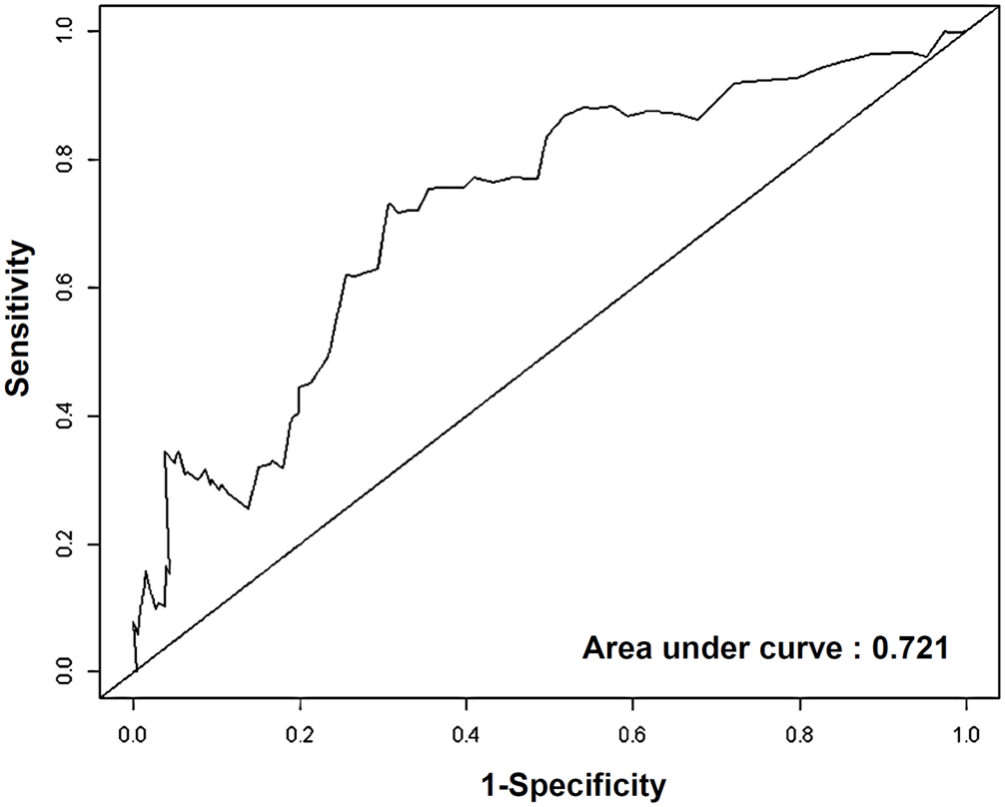
Time-dependent ROC curve for recurrence-free survival (n = 305).

**Figure 2:**
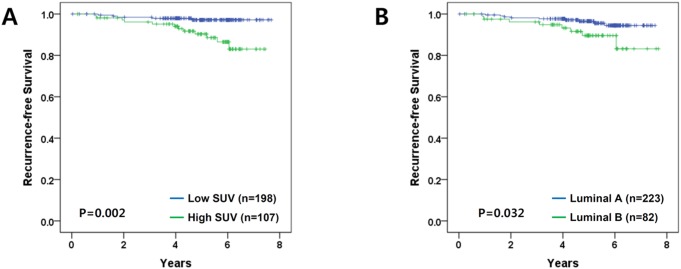
Kaplan-Meier plots for recurrence-free survival. All *P*-values are calculated by the log-rank test (A) Stratification by SUV_max_ (*P* = 0.002) (B) Stratification by the intrinsic subtypes (*P* = 0.032).

At a median follow up of 6.12 years there were 17 recurrences: 5 loco-regional and 12 distant metastases. First, we performed univariate analysis using continuous SUV_max_. This analysis showed that continuous SUV_max_ was a significant prognostic factor with a hazard ratio (HR) of 1.21 (*P* = 0.018; 95% CI, 1.03–1.42). Next, we determined the cut-off point of SUV_max_ using the time-dependent ROC curve in relation to RFS, which yielded an area under the curve (AUC) of 0.721 (95% CI, 0.594–0.884; Fig. 1). Youden’s index was highest for SUV_max_ of 4.1. With consideration of clinical application, we defined the cut-off for SUV_max_ as 4, which gave an AUC for RFS of 0.731 (95% CI, 0.592–0.902). Subsequently, high and low SUV_max_ groups were defined by a cut-off value of 4. Based on univariate analysis, the RFS time differed significantly between groups stratified by SUV_max_ (SUV_max_<4 versus≥4; *P* = 0.002; Fig. 2A).

In univariate analyses using other characteristics (Table S1), RFS differed significantly between groups stratified by age (≤35 vs. >35 years; *P*<0.001), intrinsic subtype (luminal A vs. luminal B; *P* = 0.031; Fig. 2B), and PR (positive vs. negative; *P* = 0.003).

### SUV_max_ versus intrinsic subtypes

**Figure 3:**
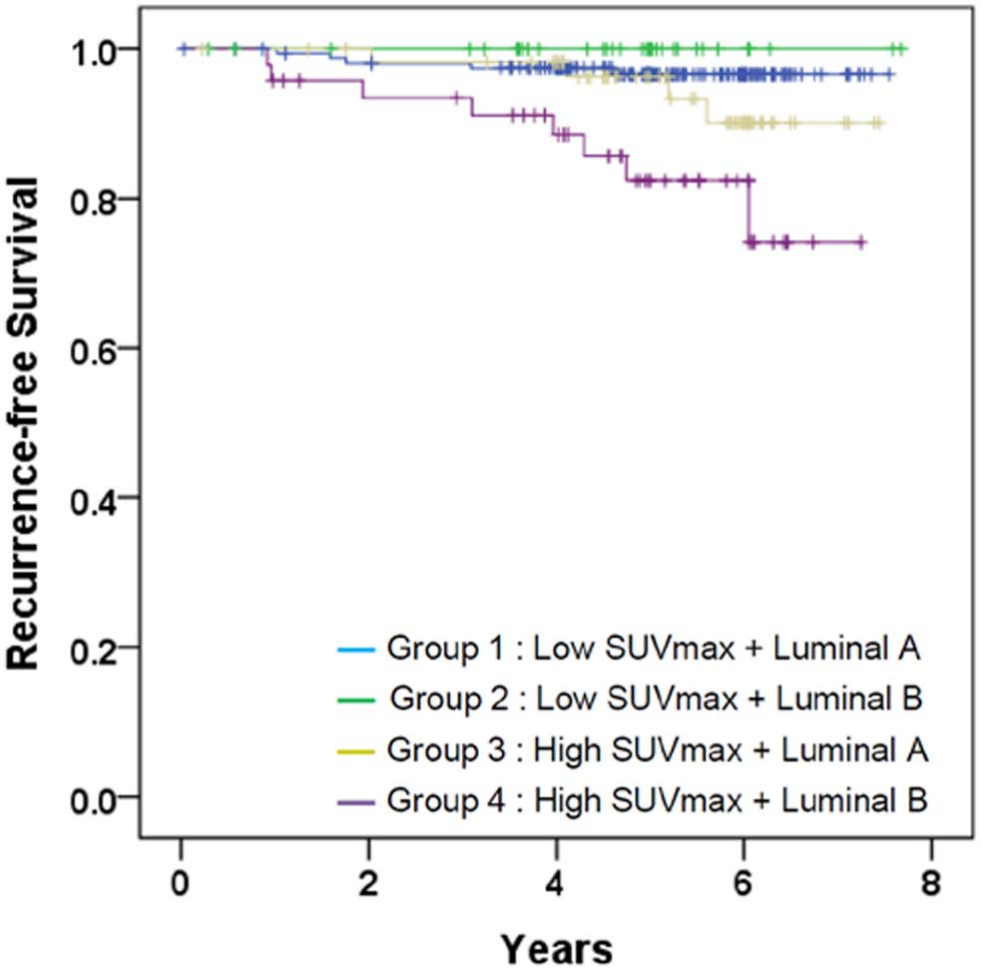
Kaplan-Meier plots stratified by combined factors with SUV_max_ and the intrinsic subtype: Group 1, low SUV_max_ and luminal A (n = 163); Group 2, low SUV_max_ and luminal B (n = 35); Group 3, high SUV_max_ and luminal A (n = 60); Group 4, high SUV_max_ and luminal B (n = 47). *P*-value calculated by the log-rank test was 0.001.

Kaplan-Meier plots comparing groups stratified by SUV_max_ and intrinsic subtype are presented in Fig. 3. RFS did not differ significantly according to subtype within the group of low SUV_max_ (group 1 vs. group 2; *P* = 0.315, log-rank test) or the group of high SUV_max_ (group 3 vs. group 4; *P* = 0.060, log-rank test). In contrast, within the group of luminal B, RFS significantly differed according to SUV_max_ (group 2 vs. group 4; *P* = 0.018, log-rank test). Within the group of luminal A, RFS did not differ significantly according to SUV_max_ (group 1 vs. group 3; *P* = 0.280, log-rank test).

### Multivariate analysis

Multivariate analysis using a Cox regression hazard model suggested that high SUV_max_ (adjusted HR 4.09; 95% CI 1.36–12.31) was an independent prognostic factor for RFS, and age less than 35 (adjusted HR 7.09; 95% CI 2.56–19.60) and negative PR status (adjusted HR 4.47; 95% CI 1.61–12.44) were associated with an increased risk of tumor recurrence (Table 2). However, the intrinsic subtype was not a prognostic factor in multivariate analysis (*P* = 0.240). For this model, the Harrell c-index was 0.729. In addition, among patients who received adjuvant chemotherapy (n = 187), dichotomized SUV_max_ was repeatedly shown to be an independent prognostic factor for RFS (adjusted HR 5.96; 95% CI 1.49–23.95; Table S2). The Harrell c-index was 0.741 in this model.

**Table 2:** Multivariate analysis using Cox regression hazard model for recurrence-free survival.

Characteristics^a^	*P*-value	Adjusted HR	95% confidence interval
**Age, years**	<0.001		
>35 (*n* = 27)		reference	
≤35 (*n* = 278)		7.09	2.56–19.60
**Progesterone receptor**	0.004		
Positive (*n* = 266)		reference	
Negative (*n* = 39)		4.47	1.61–12.44
**Intrinsic subtype**	0.274		
Luminal A (*n* = 223)		reference	
Luminal B (*n* = 82)		1.77	0.64–4.90
**SUV_max_**	0.012		
4< (*n* = 198)		reference	
≥4 (*n* = 107)		4.09	1.36–12.31

HR, hazard ratio; SUV_max_, maximum standardized uptake value.

a Presented variables are selected using Harrell *c*-statistic. In this analysis, Harrell *c*-index was 0.729.

### SUV_max_ according to tumor characteristics

The mean SUV_max_ in the two groups was compared after stratification by tumor characteristics (Table 3). The mean SUV_max_ was significantly higher for the following prognostic factors: large tumor size (*P*<0.001), positive lymph node (*P* = 0.011), positive HER2 (*P*<0.001), high Ki67 (*P* = 0.011), and high histologic grade (*P*<0.001), all of which are considered risk factors. In addition, the mean SUV_max_ of the luminal B subtype was significantly higher than that of luminal A.

**Table 3:** Comparisons of SUV_max_ according to tumor characteristics.

Characteristics	Mean SUV_max_±SD	*P*-value^a^
**Age, years**		0.224
>35 (*n* = 27)	**3.63±**2.31	
≤35 (*n* = 278)	**4.43±**3.28	
**Tumor size**		<0.001
≤2 cm (*n* = 174)	**4.72±**2.60	
>2 cm (*n* = 131)	**2.92±**1.94	
**Lymph node status**		0.011
Negative (*n* = 201)	**3.44±**2.36	
Positive (*n* = 104)	**4.18±**2.41	
**Estrogen receptor status**		0.928
Positive (*n* = 277)	**3.69±**2.43	
Negative (*n* = 28)	**3.74±**2.33	
**Progesterone receptor status**		0.394
Positive (*n* = 266)	**3.74±**2.47	
Negative (*n* = 39)	**3.43±**2.00	
**HER2**		<0.001
Negative (*n* = 258)	**3.44±**2.33	
Positive (*n* = 47)	**5.12±**2.40	
**Ki67**		0.011
<14% (*n* = 272)	**3.57±**2.35	
≥14% (*n* = 33)	**4.70±**2.76	
**Histologic grade** b		<0.001
1 and 2 (*n* = 246)	**5.85±**2.84	
3 (*n* = 26)	**3.58±**2.27	
**Subtype**		<0.001
Luminal A (*n* = 223)	**3.22±**2.15	
Luminal B (*n* = 82)	**4.98±**2.64	

HER2, human epidermal growth factor receptor-2.

a Student’s t-tests.

b Data with missing values.

## Discussion

In this genomic era, molecular profiling of breast cancer has enhanced our understanding of the complexity and heterogeneity of breast cancer [4]. To translate this understanding of intrinsic subtypes into clinical practice, IHC expression patterns have been widely investigated as surrogate markers. The experts of the St. Gallen’s panel recommended optimal guidelines for intrinsic subtyping based on IHC markers [5], [6]. In our study, we used these criteria to identify the intrinsic subtypes of HR-positive tumors.

In terms of prognostic discrimination of endocrine-responsive breast cancer, our results suggested that SUV_max_ is superior to classification based on surrogate IHC markers. SUV_max_ demonstrated independent prognostic significance in multivariate analysis whereas classification with IHC markers did not. Analysis revealed that our model including SUV_max_ had good predictive value for RFS, with a Harell *c*-index of 0.729. These findings suggest a potential role for FDG-PET in better predicting groups with poor and good prognosis among patients with HR-positive breast cancer.

It is known that HR-positive tumors show lower SUV_max_ than HR-negative tumors [10]–[12]. Regarding intrinsic subtypes, SUV_max_ was lowest in the luminal A subtype and higher in HER2 and triple negative subtypes [16], [17]. The increased accumulation of FDG observed in the high SUV_max_ tumors reflects highly proliferating and poorly differentiated cancer. Therefore, HR-positive tumors with high SUV_max_ might have aggressive behavior and show a propensity for high proliferation. Indeed, our findings suggested that SUV_max_ is associated with high Ki67, HER2 positivity, and higher histologic grade, even in HR-positive cancer (Table 3).

To evaluate tumor proliferation based on PET scans, PET imaging using 18F-fluorothymidine (FLT) has been investigated in breast cancer [18], [19]. FLT-PET is known to be closely associated with Ki67 expression in breast cancer. Although high SUV_max_ of FDG-PET correlated with high Ki67 in our study, FLT-PET is recognized as a better tool than FDG-PET in terms of evaluation of tumor proliferation. FLT-PET was not available for our study, but it will be interesting to compare the prognostic significance of FDG-PET with that of FLT-PET in HR-positive tumors.

Interestingly, in the analysis with combined factor using SUV_max_ and intrinsic subtypes (Figure 3), we noted that Group 4 had higher proportion of HER2-positive tumors than group 2 (Figure S2). As suggested in Table 3, SUV_max_ was higher in HER2-positive tumor, thus it seems reasonable that luminal B tumors with high SUV_max_ (Group 4) had a higher HER2-positive rate than luminal B tumors with low SUV_max_ (Group 2). It is concordant with previous studies that most of HER2-positive luminal B had a higher chance of tumor recurrence among hormone receptor-positive tumors [20], [21]. In addition, it is noteworthy that there might be tumors with good prognosis even in HER2-positive luminal B tumors and those tumors can be identified by SUV_max_.

Recent studies suggested that PR status should be considered as a critical prognosticator in HR-positive women [22]–[24]. Prognostic power of PR is also recognized by the panels of St. Gallen in 2013 [5]. Our data confirm the prognostic significance of PR. The reproducibility of previous findings implied that our cohort showed a reliable outcome and the quality of IHC markers was well controlled. Furthermore, our data suggest that SUV_max_ remains a good prognostic marker for HR-positive breast cancer independent of PR.

The limitations of our study reflect its retrospective nature and the heterogeneity of the patient population. One important factor is the inability to control for variations in adjuvant treatments that may influence survival outcomes. Therefore, to minimize the confounding effect of adjuvant treatment, we performed the same analyses in patients with adjuvant chemotherapy. These analyses revealed that SUV_max_ still carried prognostic significance for RFS in patients receiving both adjuvant chemotherapy and endocrine therapy.

Despite these limitations, our study highlights the prognostic impact of FDG-PET for patients with HR-positive breast cancer. The prognostic implications of SUV_max_ observed in our study warrant further investigation in prospective studies.

In conclusion, among patients with hormone receptor-positive breast cancer, FDG-PET can help discriminate patients at high risk of tumor relapse.

## Supporting Information

Figure S1 **Consort chart showing the patients identified in our study.**
(TIF)

Figure S2 **HER2-positive rates among the groups classified with IHC markers and SUV_max_.**
(TIF)

Table S1 **Univariate analyses according to tumor characteristics.**
(DOCX)

Table S2 **Multivariate analysis using Cox regression hazard model for recurrence-free survival in patients receiving adjuvant chemotherapy (n = 187).**
(DOCX)

Data S1 **Data used in this study.**
(XLS)
