# Supplementary figures and images for: [18F]-Fluorodeoxyglucose Positron Emission Tomography Can Contribute to Discriminate Patients with Poor Prognosis in Hormone Receptor-Positive Breast Cancer
Source: PLoS One. 2014 Aug 28;9(8):e105905. doi: 10.1371/journal.pone.0105905 (PMC4148332; doi:10.1371/journal.pone.0105905)

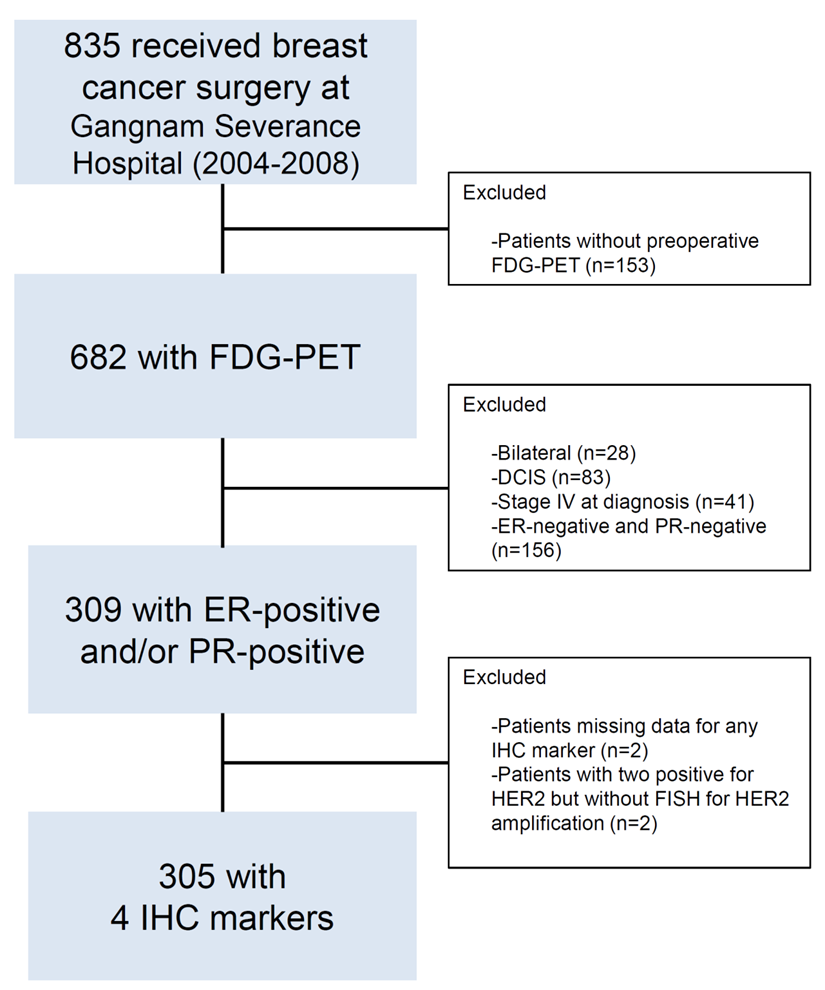

Supplement: Figure S1 — Consort chart showing the patients identified in our study. (TIF) [file pone.0105905.s001.tif]

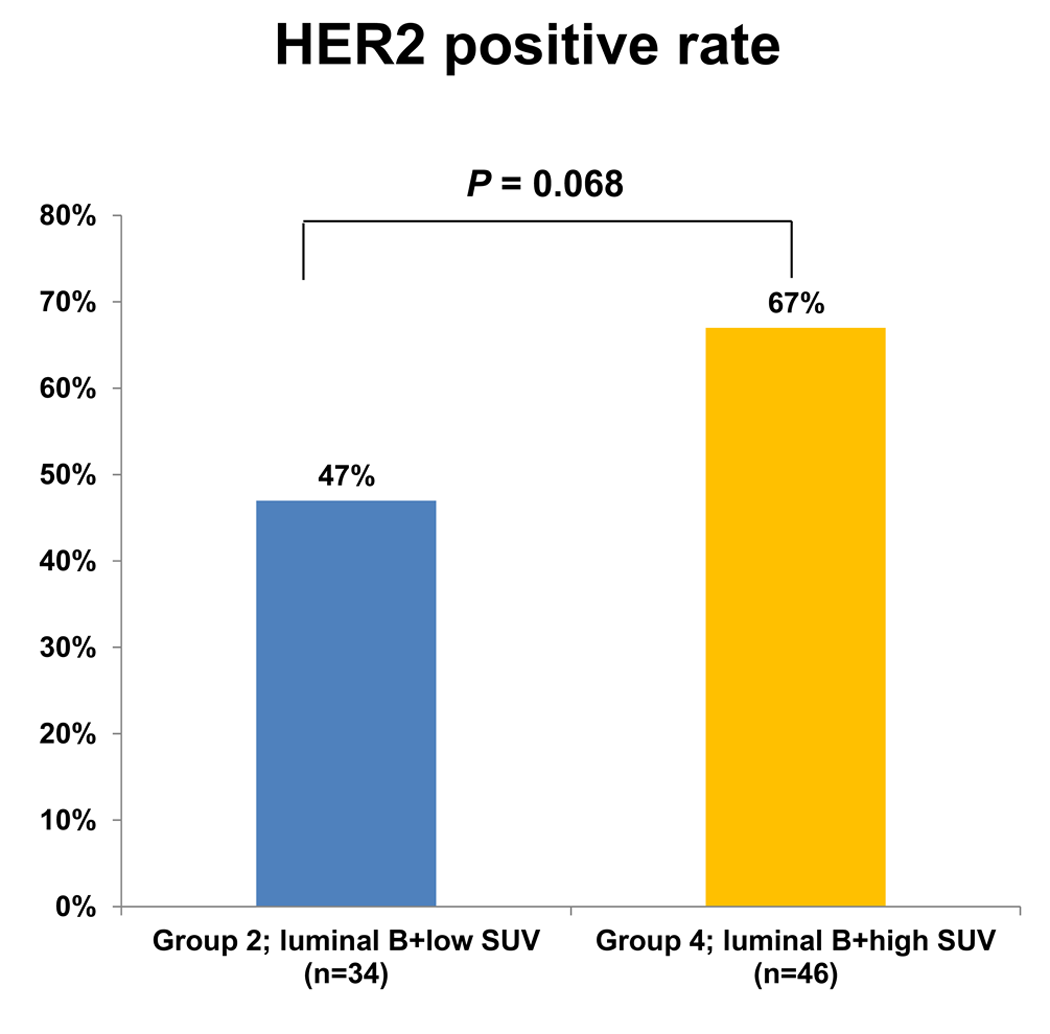

Supplement: Figure S2 — HER2-positive rates among the groups classified with IHC markers and SUVmax. (TIF) [file pone.0105905.s002.tif]
