# Supplementary material for: [18F]-Fluorodeoxyglucose Positron Emission Tomography Can Contribute to Discriminate Patients with Poor Prognosis in Hormone Receptor-Positive Breast Cancer
Source: PLoS One. 2014 Aug 28;9(8):e105905. doi: 10.1371/journal.pone.0105905 (PMC4148332; doi:10.1371/journal.pone.0105905)
Supplement: Table S1 — Univariate analyses according to tumor characteristics. (DOCX) [file pone.0105905.s003.docx]

**Table S1. Univariate analyses according to tumor characteristics**

| **Characteristics** | ***P*-value^a^** | **HR^b^** | **95% CI** |
| --- | --- | --- | --- |
| **Age** | **<0.001** | **6.27** | **2.28-17.27** |
| ≤35 (*n* = 272) vs. >35 (*n* = 27) |  |  |  |
| **T stage** | 0.439 | 1.46 | 0.56-3.78 |
| >2 cm (*n* = 174) vs. ≤2 cm (*n* = 131) |  |  |  |
| **Nodal status** | 0.109 | 2.14 | 0.83-5.55 |
| Positive (*n* = 201) vs. Negative (*n* = 104) |  |  |  |
| **AJCC stage** | 0.827 | 1.26 | 0.59-2.65 |
| I (*n* = 126) vs. II (*n* = 155) vs. III (*n* = 24) |  |  |  |
| **Histologic grades** | 0.135 | 2.53 | 0.72-8.88 |
| 1 and 2 vs. 3 |  |  |  |
| **Estrogen receptor** | 0.820 | 1.19 | 0.27-5.19 |
| Positive (*n* = 277) vs. Negative (*n* = 28) |  |  |  |
| **Progesterone receptor** | **0.003** | **4.09** | **1.51-11.07** |
| Positive (*n* = 266) vs. Negative (*n* = 39) |  |  |  |
| **HER-2** | 0.105 | 2.32 | 0.82-6.57 |
| Negative (*n* = 258) vs. Positive (*n* = 47) |  |  |  |
| **Ki67** | 0.710 | 1.32 | 0.30-5.79 |
| Low (*n* = 272) vs. High (*n* = 33) |  |  |  |
| **Subtypes** | **0.031** | **2.20** | **1.01-5.84** |
| Luminal A (*n* = 223) vs. Luminal B (*n* = 82) |  |  |  |
| **Dichotomized SUV_max_** | **0.002** | **4.38** | **1.54-12.43** |
| Low (*n* = 198) vs. High (*n* = 107) |  |  |  |
| **Adjuvant endocrine therapy** | 0.732 | 0.81 | 0.52-1.27 |
| Tamoxifen (*n* =129) vs. Toremifene (*n* = 62) vs. Anastrozole (*n* = 61) vs. Letrozole (*n* = 53) |  |  |  |
| **Adjuvant chemotherapy** | 0.564 | 1.33 | 0.50-3.50 |
| Yes (*n* = 187) vs. No (*n* = 118) |  |  |  |
| **Adjuvant radiotherapy** | 0.947 | 1.03 | 0.39-2.72 |
| Yes (*n* = 119) vs. No (*n* = 186) |  |  |  |

HR, hazard ratio; HER2, human epidermal growth factor receptor-2; SUV_max_, maximum standardized uptake value.

^a^ The log-rank test

^b^ HR was calculated using Cox regression hazard model.
