# Supplementary material for: [18F]-Fluorodeoxyglucose Positron Emission Tomography Can Contribute to Discriminate Patients with Poor Prognosis in Hormone Receptor-Positive Breast Cancer
Source: PLoS One. 2014 Aug 28;9(8):e105905. doi: 10.1371/journal.pone.0105905 (PMC4148332; doi:10.1371/journal.pone.0105905)
Supplement: Table S2 — Multivariate analysis using Cox regression hazard model for recurrence-free survival in patients receiving adjuvant chemotherapy (n = 187). (DOCX) [file pone.0105905.s004.docx]

**Table S2. Multivariate analysis using Cox regression hazard model for recurrence-free survival in patients receiving adjuvant chemotherapy (*n* = 187)**

| **Characteristics^a^** | ***P*-value** | **Adjusted HR** | **95% confidence interval** |
| --- | --- | --- | --- |
| **Age** | < 0.001 |  |  |
| >35 (*n* = 22) |  | reference |  |
| ≤35 (*n* = 165) |  | 14.91 | 3.71–59.91 |
|  |  |  |  |
| **Progesterone receptor** | 0.012 |  |  |
| Positive (*n* = 164) |  | reference |  |
| Negative (*n* = 23) |  | 5.96 | 1.49–23.95 |
|  |  |  |  |
| **Intrinsic subtype** | 0.172 |  |  |
| Luminal A (*n* = 107) |  | reference |  |
| Luminal B (*n* = 80) |  | 2.66 | 0.65–10.85 |
|  |  |  |  |
| **SUV_max_** | 0.027 |  |  |
| <4 (*n* = 131) |  | reference |  |
| ≥4 (*n* = 56) |  | 11.76 | 1.32–105.07 |

HR, hazard ratio; SUV_max_, maximum standardized uptake value.

^a^ Presented variables were selected using Harrell *c*-statistic. In this analysis, Harrell *c*-index was 0.741
